# Emergency Tracheal Intubation in Patients with COVID-19: Experience from a UK Centre

**DOI:** 10.1155/2020/8816729

**Published:** 2020-12-10

**Authors:** Ajay Gandhi, Jagdish Sokhi, Chris Lockie, Patrick A. Ward

**Affiliations:** Chelsea and Westminster Hospital, London, UK

## Abstract

This retrospective observational case series describes a single centre's preparations and experience of 53 emergency tracheal intubations in patients with COVID-19 respiratory failure. The findings of a contemporaneous online survey exploring technical and nontechnical aspects of airway management, completed by intubation team members, are also presented. Preparations included developing a COVID-19 intubation standard operating procedure and checklist, dedicated airway trolleys, a consultant-led mobile intubation team, and an airway education programme. Tracheal intubation was successful in all patients. Intubation first-pass success rate was 85%, first-line videolaryngoscopy use 79%, oxygen desaturation 49%, and hypotension 21%. Performance was consistent across all clinical areas. The main factor impeding first-pass success was larger diameter tracheal tubes. The majority of intubations was performed by consultant anaesthetists. Nonconsultant intubations demonstrated higher oxygen desaturation rates (75% vs. 45%, *p*=0.610) and lower first-pass success (0% vs. 92%, *p* < 0.001). Survey respondents (*n* = 29) reported increased anxiety at the start of the pandemic, with statistically significant reduction as the pandemic progressed (median: 4/5 very high vs. 2/5 low anxiety, *p* < 0.001). Reported procedural/environmental challenges included performing tasks in personal protective equipment (62%), remote-site working (48%), and modification of normal practices (41%)—specifically, the use of larger diameter tracheal tubes (21%). Hypoxaemia was identified by 90% of respondents as the most challenging patient-related factor during intubations. Our findings demonstrate that a consultant-led mobile intubation team can safely perform tracheal intubation in critically ill COVID-19 patients across all clinical areas, aided by thorough preparation and training, despite heightened anxiety levels.

## 1. Introduction

The coronavirus disease 19 (COVID-19) pandemic has resulted in more than 6.25 million confirmed cases and 375,000 deaths across 215 countries [1, 2]. In March 2020, there was a rapid upsurge in critically ill patients diagnosed with severe acute respiratory syndrome coronavirus 2 (SARS-CoV-2) requiring emergency tracheal intubation at our institution [3–5]. Tracheal intubation in these patients poses a unique set of challenges. It combines complex time-critical tasks in physiologically difficult airways, with heightened clinician anxieties relating to personal protective equipment (PPE) and health risk from viral exposure associated with aerosol-generating procedures (AGPs) [6]. Increased anxiety levels have been reported in clinicians relating to higher mortality rates in healthcare workers [7–9]. The potential impact of these increased anxiety levels upon airway management has not yet been investigated [3]. Recognising that situational awareness, decision-making, and team performance during tracheal intubation may be negatively affected by clinician anxiety, we rapidly modified our standard approach to emergency airway management in critically ill patients, implementing a comprehensive dedicated COVID-19 airway management strategy.

In this case series, we outline our local airway management preparations and subsequent experience of emergency tracheal intubation in COVID-19 patients. We aimed to compare the incidence of complications and first-pass success rate at our institution with internationally reported rates in COVID-19 patients. We also make comparison with historical emergency airway intervention outcome data in non-COVID-19 patients previously managed at our institution. In addition, we explored technical and nontechnical aspects of airway management using an online survey, completed by intubation team members, specifically investigating factors contributing towards anxiety.

## 2. Methods

### 2.1. Preparation Phase

Our preparations preceded official guidance from UK national societies, such as the Intensive Care Society and Difficult Airway Society [10]. Anticipating the surge in numbers and significant challenges associated with emergency tracheal intubation in COVID-19 patients, we reviewed the available literature, synthesising early airway management experiences from Wuhan, China, and Lombardy, Italy, with international expert opinion [3, 11–13], to develop and implement a comprehensive airway management strategy.

### 2.2. Standardised Technique and Equipment

A COVID-19 intubation standard operating procedure (SOP) and checklist were devised (1 in Supplementary Materials). Mobile COVID-19 intubation trolleys, mounted with equipment shadow boards and containing standardised equipment in a uniform configuration, were created. Videolaryngoscopy (GlideScope®, Verathon Medical, Bothell, WA) was recommended as first-line for tracheal intubation. A second-generation supraglottic airway device (SAD) was recommended for first-line rescue oxygenation. “Grab bags” were prepared, containing enhanced PPE for AGPs, comprising a surgical hat, fit-tested FFP3 respirator mask, full-face visor, long-sleeved fluid-resistant gown, and two pairs of surgical gloves.

### 2.3. Intubation and PPE Training

The intubation checklist and SOP were demonstrated using high-fidelity simulation, live streamed to all relevant staff, complemented by lectures, and small-group in situ simulation. Staff were provided with hands-on training and given the opportunity to practice tracheal intubation (on manikins) using the new checklist, SOP, and airway trolley to ensure familiarisation, competence, and knowledge retention. Practical tips on overcoming communication difficulties whilst wearing PPE and techniques to minimise potential aerosol generation were shared, as well as highlighting the importance of thorough preparation. Comprehensive training in PPE use was provided, with small group workshops conducted with experienced trainers, affording staff the opportunity to observe live demonstration of safe doffing and donning procedures and then to practice the technique with instructions. All sessions were made available via an open-access online resource.

### 2.4. Intubation Teams

Anaesthesia consultants and trainees were redeployed to critical care to meet the clinical demand. A mobile intubation team rota was established, ensuring two consultant anaesthetists and one operating department practitioner (ODP) at every emergency intubation. A dedicated handover document (2 in Supplementary Materials) was employed to guarantee a structured process during shift changes, ensuring clear allocation of team roles and highlighting high-risk patients/those anticipated to require tracheal intubation. Communication between team members was also facilitated by encrypted WhatsApp group and easy-clean walkie-talkies for use in contaminated areas.

### 2.5. Data Collection Phase

#### 2.5.1. Tracheal Intubation Database

This retrospective observational case series was undertaken at a single site, Chelsea and Westminster Hospital, London, UK. All adult patients diagnosed with COVID-19, requiring tracheal intubation between March 13 and May 1, 2020, were included. Following each intubation, the intubation team documented location, team composition, patient demographics and evaluations (physiological status, airway, and comorbidities), airway management details (indication, grade, technique, equipment, number of attempts, and rescue oxygenation), and immediate complications (aspiration, oxygen desaturation, hypotension, cardiorespiratory arrest, and PPE issues). Data were retrospectively collected from the hospital electronic patient record system by two investigators (AG and JS) for accuracy.

#### 2.5.2. Online Survey

A multiple-choice survey (3 in Supplementary Materials) was circulated to all intubation team clinicians from May 7, 2020, with all survey responses collated by May 14, 2020. Survey questions examined technical and nontechnical aspects of their airway management experience, including factors influencing performance and perceived anxiety (scored using Likert scales). Free-text responses were encouraged. Respondents could only complete the survey once, and data were anonymised.

#### 2.5.3. Ethics

Data were extracted and anonymised in accordance with the internal information governance review, NHS Trust information governance approval, and Caldicott Guardian procedures outlined under the Strategic Research Agreement. No specific research and ethics committee approval was required.

#### 2.5.4. Statistical Analysis

Post hoc statistical analysis was performed using SPSS® version 22, IBM®, Chicago, IL, USA. Numerical data were assessed for normality using the Shapiro–Wilk test. Comparisons of outcomes in relation to categorical variables have been described using chi-squared or Fisher's exact tests. All reported *p* values are one-sided and are considered to be statistically significant if *p* < 0.05.

## 3. Results

### 3.1. Intubation Data

#### 3.1.1. Inclusion Criteria and Demographics

During the study period, 52 patients underwent tracheal intubation (median age: 57 years; IQR: 53.0–67.0; 77% male; Table 1). All patients were confirmed as SARS-CoV-2 positive on polymerase chain reaction testing. One patient failed tracheal extubation, requiring reintubation (after >24 hours of continuous positive airway pressure (CPAP) ventilation), yielding a total of 53 intubations.

#### 3.1.2. Patient Comorbidities

The principal comorbidity was cardiovascular diseases (47%): hypertension 36%; atrial fibrillation 8%; ischaemic heart disease 4%. High body mass index (BMI) > 30 kg/m^2^ (38%), respiratory disease—asthma or chronic obstructive pulmonary disease (36%), and diabetes (26%) were also prevalent (Table 1).

#### 3.1.3. Respiratory Support

Prior to intubation team involvement, 38% of patients received high-flow oxygen via a non-rebreathe face mask, and 60% received CPAP (Table 1). The indication for intubation was COVID-19 respiratory failure in all patients, with increased work of breathing or profound hypoxia present in all. The median SpO_2_/FIO_2_ (SF) ratio was 96 (IQR: 92–119). The SF ratio was utilised rather than the more widely recognised PaO_2_/FIO_2_ (PF) ratio as arterial blood gas analysis was not undertaken in all patients prior to tracheal intubation. The SF ratio is a validated, noninvasive, surrogate for the PF ratio.

#### 3.1.4. Clinical Location

Two-thirds of intubations were undertaken in remote clinical areas (Table 2), with 38% occurring in the emergency department (ED) and 28% on medical wards. Less commonly, intubations were undertaken in nonremote sites (operating theatres or intensive care unit (ICU)).

#### 3.1.5. Team Composition and PPE

Intubation teams consistently comprised (i) primary intubator, (ii) ODP, (iii) team leader/secondary intubator, (iv) clinician responsible for drug administration/monitoring, and (v) spare assistant (if available). In 90% of intubations, there were 4 or 5 team members, all present in the room and all wearing enhanced PPE. There were no reported PPE breaches or intubation difficulties directly relating to PPE.

#### 3.1.6. Airway Management

All patients underwent successful tracheal intubation with cuffed tracheal tubes with subglottic suction ports (Portex®, Smiths Medical, USA). First-pass intubation success rate was 85% (Table 2). A second (or more) intubation attempt was required in 15% (8/53), with 6/8 requiring (successful) rescue oxygenation via a SAD (SpO_2_ > 90%). No patients received bag-mask ventilation for rescue oxygenation (Table 3). Reasons for repeat intubation attempts included difficulty in passing larger diameter tracheal tubes (5/8), glottic secretions (2/8), and inadequate view requiring change in the videolaryngoscope blade (1/8). There was no significant difference between first-pass success rates in remote versus nonremote sites, reported as 89% and 78%, respectively (*p*=0.26) (Table 4).

All patients were preoxygenated for ≥3 minutes using a two-handed, tight-fitting face mask technique, as per the intubation SOP. All patients underwent tracheal intubation via modified rapid sequence induction (RSI). Cricoid pressure was applied in 42%. No patients underwent bag-mask ventilation during the apnoea window. Apnoeic oxygenation was provided via a tight-fitting face mask. Nasal cannula per-oxygenation was not used.

Videolaryngoscopy was used in 79% of intubations. All intubations were performed by anaesthetists or intensivists, and the primary intubator was a consultant in 92% (Table 4). Remaining intubations were undertaken by senior trainees, with immediate consultant supervision. All of these (4/4) were unsuccessful on the first attempt. Consultant and trainee first-pass success rates were significantly different (92% versus 0%, respectively, *p* < 0.001) (Table 4).

### 3.2. Induction Agents

Induction drugs were recorded in 96% (51/53) of patients (Table 3). Fentanyl was used in 94% of cases (median dose: 2.5 mcg/kg; IQR: 0.66), propofol in 87% of cases (median dose: 1.22 mg/kg; IQR: 0.7), and rocuronium in 94% of cases (median dose: 1.20 mg/kg; IQR: 0.27).

### 3.3. Complications

Oxygen desaturation (SpO_2_ < 90%) occurred in 49% of intubations, with greater frequency in those undertaken by trainees (75% (3/4)) compared with consultants (47% (23/49)). Desaturations occurred more commonly in patients requiring multiple attempts compared with first-pass success (63% versus 19%, *p* < 0.007). Use of CPAP prior to intubation did not confer a statistically significant effect on desaturation rates when compared with high-flow oxygen therapy (Table 4). Incidence of desaturation was consistent across all clinical areas (Table 4).

Regurgitated gastric contents (low volume, nonparticulate) were noted at intubation in 4% (2/53) of patients, though none was clinically significant. Cricoid pressure had been applied in both patients (Table 3).

No pneumothoraces were demonstrated after intubation. No emergency front-of-neck airways (eFONA) were performed. No hypoxic respiratory arrests occurred.

Clinically significant hypotension (systolic blood pressure<90 mmHg) occurred in 21% (11/53) of patients. No cardiac arrests occurred during or immediately after intubation (Table 3).

### 3.4. Survey Data

#### 3.4.1. Demographics and Inclusion Criteria

The survey was circulated to all 40 intubation team clinicians. 29 surveys were completed (73% response rate), of whom 79% (23/29) were consultants, with the remainder being senior anaesthetic trainees (Table 5). 90% (26/29) had been primary intubator in at least one intubation, with the majority performing 1-2 intubations (median: 1.5; IQR: 4). 86% of respondents reported videolaryngoscopy use in <50% of pre-COVID-19 intubations (Table 5). Only 7% reported routine use in >75% intubations.

**Figure 1:**
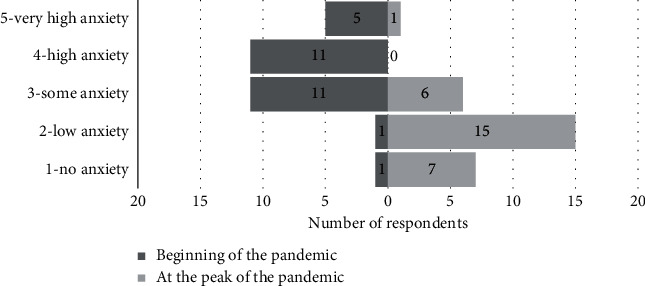
Changes in perceived anxiety levels reported by clinicians involved in emergency tracheal intubations over the course of the pandemic (*n* = 29).

Respondents were asked to grade their perceived degree of anxiety at the onset of the pandemic, when the first intubations were undertaken (1 = no anxiety; 5 = very high anxiety). Median perceived anxiety levels were 4/5 (IQR: 1), with 96% (27/29) reporting intermediate-to-high levels (Table 6). Respondents were asked to grade their perceived degree of anxiety at the pandemic peak (highest frequency of intubations). Median anxiety levels were 2/5 (IQR: 0.5), demonstrating a significant reduction (*p* < 0.001) (Figure 1).

Respondents were asked to identify environmental/procedural factors contributing to anxiety. Stressors most commonly identified included performing tasks in PPE (62%), remote-site working (48%), and modification of normal practices (41%). Larger diameter tracheal tubes (with subglottic suction ports) were identified as a technical challenge (21%), along with working in unfamiliar teams (14%), the intubation checklist (7%), first-line use of videolaryngoscopy (7%), and SAD for rescue oxygenation (3%). Respondents were asked to identify patient-related factors contributing to intubation difficulty, with 90% identifying severe hypoxaemia. Use of CPAP (31%), high BMI (31%), and increased airway oedema (21%) were also identified. Respondents were asked to identify aspects of PPE that were most challenging during intubations, with 93% reporting difficulty hearing/impaired communication, 52% reduced vision, and 48% temperature (Table 6).

## 4. Discussion

This case series demonstrates that a consultant-led team can safely undertake emergency tracheal intubation in COVID-19 patients with hypoxic respiratory failure, incurring relatively few major complications. The local airway management strategy which we rapidly implemented was reassuringly consistent with subsequent guidance from UK national professional bodies [10]. Despite significant clinician anxiety at the onset of the pandemic, performance was consistently high across all clinical areas, facilitated by thorough preparation, standardised practice, and the use of cognitive aids, contributing to a reduction in perceived anxiety as the pandemic progressed.

### 4.1. Demographics and Comorbidities

Our institution is a busy district general hospital in central London with a large ED, servicing a diverse ethnic and socioeconomic population. The prevalence of pre-existing comorbidities (especially, the predominance of cardiovascular diseases and higher BMI), average age, and male gender preponderance is consistent with international datasets [3, 14–17]. The preintubation SF ratios reflect a degree of hypoxia in keeping with severe acute respiratory distress syndrome (ARDS) [18–20], suggesting our sample is representative.

### 4.2. Airway Management

The first-pass intubation success rate of 85% is consistent with internationally reported rates in COVID-19 patients [3] and comparable to in-hospital and prehospital first-pass rates in non-COVID-19 critically unwell adults [21]. When compared with our hospital's pre-COVID-19 ICU intubation data (biannual audit, completed January 2020), there were improved first-pass success (85% versus 74%) and increased videolaryngoscopy use (87% versus 41%) in the COVID-19 intubations, likely reflective of increased consultant intubators (92% versus 10%) and our intubation SOP recommendations. All COVID-19 intubations undertaken by nonconsultants required a second attempt. These findings support our recommendation that the most experienced airway-trained practitioner should be responsible for airway management and the requirement for a consultant intubation rota.

Our intubation checklist and SOP were robust in practice, facilitating consistently high performance in stressful situations, in high-risk patients, in remote clinical areas. Standardising practice and the use of cognitive aids are well recognised in relieving cognitive burden and improving team performance in high-stress situations [22]. First-pass success rates were consistently high across the pandemic, and a significant reduction in perceived anxiety was reported. This may reflect increased familiarity with the intubation process, within teams, with PPE and in managing COVID-19 patients.

Videolaryngoscopy has been widely advocated in this population, to increase the first-pass success rate and to maximise the patient-operator distance, forming a key component of our SOP [1, 12, 23–25]. For those less versed in videolaryngoscopy, we recommended using their most familiar technique. Videolaryngoscopy was employed in the majority of our intubations; therefore, it was surprising that the survey reported such infrequent pre-COVID-19 routine videolaryngoscopy use. This suggests that videolaryngoscopy was adopted safely and effectively by those potentially less familiar with the technique.

Larger diameter tracheal tubes, in the presence of potentially increased airway oedema in COVID-19 patients, may contribute to difficulties with tracheal intubation and extubation. Tube size was identified as a contributory factor in 63% of intubations requiring multiple attempts, suggesting that either initial tube selection was poor, subglottic suction tube diameter was underestimated, or tube passage was impeded by glottic oedema [26]. Larger diameter tubes and airway oedema were also highlighted in survey responses as contributory factors in intubation difficulties. Although larger tracheal tubes with subglottic suction ports are generally recommended for patients with severe respiratory failure [27], particular consideration of tube size in the context of glottic oedema is warranted.

### 4.3. Complications

Given the degree of physiological derangement reflected in preintubation SF ratios, oxygen desaturation was predictably common. Though less than internationally reported rates [3], desaturation was markedly higher in comparison with our pre-COVID-19 ICU intubations (49% versus 25%, biannual audit). None of the patients underwent bag-mask ventilation during the apnoea window (due to concerns over aerosol generation), and this represents deviation from our standard practice, where gentle bag-mask ventilation is initiated if desaturation occurs [22]. Patients received some apnoeic oxygenation via a tight-fitting face mask, and we report 100% successful rescue oxygenation via SAD, with no eFONA cases. Nevertheless, an alternative method of per-oxygenation (e.g., low flow via nasal cannulae) may have reduced the incidence of desaturation. No pneumothoraces were identified, despite reported international incidence as high as 5.9% [3, 28].

Other institutions reported significant rates of peri-intubation hypotension and cardiac arrest [3, 29–31]. However, our case series demonstrated relative cardiovascular stability, with no cardiac arrests and hypotension rates consistent with pre-COVID-19 ICU intubations (18% hypotension, biannual audit). This may be partly due to our recommended induction regimen, consisting of high-dose opioid and significantly restricted propofol dosages [3, 32, 33].

### 4.4. Respiratory Support

**Figure 2:**
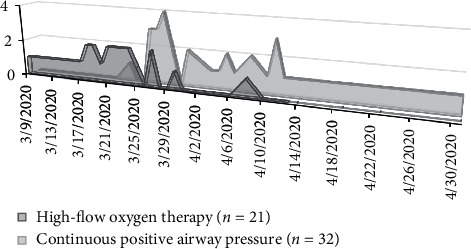
Changes in the mode of oxygen therapy delivered to the patient prior to the arrival of the intubation team over the course of the pandemic (high-flow oxygen therapy, *n* = 21/53; continuous positive airway pressure, *n* = 32/53).

Survey respondents identified hypoxaemia as the major patient-related factor contributing to anxiety. Early recommendations advocated prompt intubation with a limited temporising role for CPAP or high-flow nasal cannulae due to concerns over aerosol generation and potentiation of existing lung injury [34–37]. However, in keeping with evolving international trends [38], CPAP became more widely utilised in our patients as the pandemic progressed (Figure 2). The use of CPAP was identified as the second largest anxiety-inducing patient-related factor by survey respondents, possibly relating to the risk of aerosol generation and restricted airway access. Interestingly, there was no difference in desaturation rates between those on CPAP or conventional oxygen therapy.

### 4.5. Intubation Team and Location

Mobile intubation teams have been employed in the management of COVID-19 patients [1, 12]. Ideal team size is unknown, balancing staff viral exposure with providing the necessary flexibility and skill mix to achieve optimum outcomes. Generally consistent with other institutions, we determined a 4- or 5-person team was desirable, although 3-person teams had been described [5]. We believe our highly experienced team, staffed by a dedicated consultant anaesthetist rota, contributed to our relatively low complication rate. Working in unfamiliar teams in high-stress situations may lead to impaired team function, and this was recognised in the survey responses. Despite these challenges, performance was consistent across the relatively large number of different clinicians implementing our airway strategy, in the role of primary intubator.

Differences in the rates of oxygen desaturation and first-pass success between consultants and senior trainees reinforce our SOP recommendation (and consensus statements [10]) that the most experienced airway-trained practitioner should assume the role of the primary intubator in these challenging patients. It must be acknowledged that only a small proportion of intubations were performed by nonconsultants, prohibiting any task-repetition improvement.

Limitations of this case series include the single-centre design, retrospective data collection, and modest sample size. Multicentre projects are generally more desirable. Whilst our patient characteristics were similar to those in other case series, our resources and staff skill mix may not be representative. The online survey was not formally validated and was performed after the study period; therefore, questions relating to prepandemic anxiety levels may suffer from recall bias. We did not specifically examine the relative effectiveness of PPE or COVID-19 transmission. Other studies, to which we have contributed, may offer insight into this area [6]. Our aim was to report immediate complications; however, further work should focus on the effect of complications on longer-term patient outcomes, including mortality.

From our experience, we make the following recommendations:

1. Institutional preparedness (early introduction of checklists [39], SOPs, equipment standardisation, and staff education) is essential in reducing cognitive load and anxiety and optimising team performance
2. First-pass tracheal intubation success should be maximised by the most experienced airway-trained practitioner, assuming the role of the primary intubator, in combination with videolaryngoscopy and judicious tracheal tube selection
3. Cardiovascular stability at induction can be achieved with a high-fentanyl, low-propofol regimen
4. Gentle bag-mask ventilation or nasal cannulae “per-oxygenation” (during the apnoeic period following neuromuscular blockade) may be considered to reduce oxygen desaturation

This case series has demonstrated that our consultant-led mobile intubation team has safely performed tracheal intubations in critically unwell COVID-19 patients across multiple clinical areas. Despite clinicians reporting significant patient, environmental, and procedural challenges and heightened anxiety relating to these, intubations were all conducted successfully with relatively few major complications. We have shown that thorough preparation and training, centred on a robust SOP and checklist, can contribute to high performance and reduced anxiety in teams of clinicians managing an extremely challenging group of patients.

## Figures and Tables

**Table 1:** Baseline patient characteristics prior to tracheal intubation. Data are expressed as *n*/*N* (%) or median with interquartile range (IQR).

*Patient demographics*	
Age	57 (53–67)
Male gender	41/53 (77%)

*Patient premorbid medical conditions*	
Cardiovascular diseases	25/53 (47%)
Hypertension	19 (36%)
Atrial fibrillation	4 (8%)
Ischaemic heart disease	2 (4%)
Obesity (BMI>30 kg/m^2^)	20/53 (38%)
Respiratory disease	19/53 (36%)
Diabetes	14/53 (26%)

*Mode of oxygen delivery prior to intubation team arrival*	
Continuous positive airway pressure	32/53 (60%)
<2 days	18/53 (34%)
>2 days	15/53 (28%)
High-flow oxygen therapy	20/53 (38%)
Non-rebreathe mask	19/53 (36%)
Nasal cannulae	1/53 (2%)

*Degree of hypoxia prior to intubation team arrival*	*SpO*_*2*_ * /FIO*_*2*_ * ratio*
All patients (*N* = 53)	96 (92–119)
Continuous positive airway pressure <2 days (18/53)	96 (92–110)
Continuous positive airway pressure >2 days (15/53)	95 (92–119)
High-flow oxygen therapy (20/53)	101 (94–133)

**Table 2:** Tracheal intubation data. Data are expressed as *n*/*N* (%) or median and interquartile range (IQR).

*Location of tracheal intubation*	

Remote	35/53 (66%)
Emergency department	20 (38%)
General medical ward	15 (28%)

Nonremote	18/53 (34%)
Intensive care unit	9 (17%)
Operating theatres	9 (17%)

*Primary intubator/laryngoscopist*	

Consultant	49/53 (92%)
Senior trainee	4/53 (8%)

*Number of tracheal intubation attempts*	

One	45/53 (85%)
Two	7/53 (13%)
Three	1/53 (2%)
Failed	0/53 (0%)

*Laryngoscopy technique*	

Direct laryngoscopy	4/53 (8%)
Videolaryngoscopy	42/53 (79%)
Not specified	7/53 (13%)
Stylet	45/53 (85%)
Bougie	6/53 (11%)
Adjunct not required	2/53 (4%)
Cricoid pressure	22/53 (42%)

*Induction drugs*	

Induction agent	
Propofol	46/53 (87%)
Ketamine	4/53 (8%)
Thiopentone	1/53 (2%)
Not specified	2/53 (4%)

Median dose of commonly used drugs	
Fentanyl 94% (50/53)	2.5 mcg/kg (IQR 0.66)
Propofol 87% (46/53)	1.22 mg/kg (IQR 0.7)
Rocuronium 94.3% (50/53)	1.20 mg/kg (IQR 0.27)

**Table 3:** Incidence of complications at tracheal intubation. Data are expressed as *n*/*N* (%).

Complications at tracheal intubation	
Oxygen desaturation (SpO_2_ < 90%)	26/53 (49%)
Rescue oxygenation using the supraglottic airway device	6/53 (11%)
Rescue oxygenation using bag-mask ventilation	0/53 (0%)
Hypotension (systolic blood pressure < 90 mmHg)	11/53 (21%)
Cardiorespiratory arrest	0/53 (0%)
Pneumothorax	0/53 (0%)
Regurgitation of the gastric fluid	2/53 (4%)

**Table 4:** Factors affecting incidence of oxygen desaturation and tracheal intubation success. Data are expressed as *n*/*N* (%).

Factors affecting incidence of oxygen desaturation	Incidence of oxygen desaturation (SpO_2_ < 90%)	*p* value
Grade of the primary intubator		
Consultant	23/49 (47%)	
Senior trainee	3/4 (75%)	*p*=0.610
Preintubation oxygen therapy		
CPAP	15/32 (47%)	
No CPAP	11/21 (52%)	*p*=0.456
Number of intubation attempts		
One attempt	19/45 (42%)	
Multiple attempts	7/8 (88%)	*p*=0.022^*∗*^
Intubation location		
Remote site	16/35 (46%)	
Nonremote site	10/18 (56%)	*p*=0.349

Factors affecting success at tracheal intubation	Incidence of first pass success	*p* value

Grade of the primary intubator		
Consultant (*n* = 49)	45/49 (92%)	
Senior trainee (*n* = 4)	0/4 (0%)	*p* < 0.001^*∗*^
Phase of the pandemic		
Epoch 1 (first 18 intubations)	14/18 (78%)	vs. epoch 1
Epoch 2 (next 18 intubations)	16/18 (89%)	*p*=0.329
Epoch 3 (final 17 intubations)	16/17 (94%)	*p*=0.358
Intubation location		
Remote site	31/35 (89%)	
Nonremote site	14/18 (78%)	*p*=0.260

**Table 5:** Summary of survey responses. Data are expressed as *n*/*N* (%) or median and interquartile range (IQR).

*Role of the respondent*			
Anaesthetic consultant	18/29 (62%)		
Critical care consultant	5/29 (17%)		
Anaesthetics trainee	6/29 (21%)		

*Reported routine use of videolaryngoscopy (prepandemic)*			
<50% of tracheal intubations	25/29 (86%)		
50–75% of tracheal intubations	2/29 (7%)		
>75% of tracheal intubations	2/29 (7%)		

*Number of tracheal intubations undertaken as primary intubator/laryngoscopist*			
<2	15/29 (56%)		
3-4	6/29 (23%)		
5-6	4/29 (15%)		
7-8	2/29 (8%)		
9-10	1/29 (4%)		
>10	1/29 (4%)		

*Median (IQR)*	*1.5 (4)*		

*Perceived anxiety associated with the tracheal intubation process*	*Onset of pandemic*	*During peak of pandemic*	*p value*
Median (IQR)	4 (1)	2 (0.5)	*p* < 0.001

**Table 6:** Factors affecting perceived anxiety associated with tracheal intubations, from online survey. Data are expressed as *n*/*N* (%).

*Procedural and environmental factors*	

Personal protective equipment	18/29 (62%)

Remote location	14/29 (48%)

Performance anxiety	7/29 (24%)

Unfamiliar team	4/29 (14%)

Technical aspect	12/29 (41%)
Larger diameter tracheal tube	6/29 (21%)
Adapted/modified technique	6/29 (21%)
Primary use of videolaryngoscopy	2/29 (7%)
Use of intubation checklist	2/29 (7%)
Use of supraglottic airway for rescue ventilation	1/29 (3%)

*Factors relating to personal protective equipment*	

Hearing/communication	27/29 (93%)

Vision	15/29 (52%)

Temperature	14/29 (48%)

Physical	9/29 (31%)

*Patient factors*	

Hypoxaemic patient	26/29 (90%)

Presence of continuous positive airway pressure	9/29 (31%)

High body mass index	9/29 (31%)

Difficulties in optimising patient positioning	7/29 (24%)

Concerns over airway oedema	6/29 (21%)

Concerns over cardiovascular instability	3/29 (10%)

## Data Availability

The data used to support the results of this study were drawn from an anonymised local hospital tracheal intubation database, maintained by the authors, and the data are included within the article itself. The data are presented in tables and figures throughout the main body of the manuscript. The data are available from the corresponding author upon request for researchers who meet the criteria for accessing confidential data.

## Supplementary Materials

Supplementary Materials (1) COVID-19 intubation checklist, (2) an intubation team handover document, and (3) Chelsea COVID-19 intubation experience.
