# Supplementary material for: Emergency Tracheal Intubation in Patients with COVID-19: Experience from a UK Centre
Source: Anesthesiol Res Pract. 2020 Dec 10;2020:8816729. doi: 10.1155/2020/8816729 (PMC7729388; doi:10.1155/2020/8816729)
Supplement: Supplementary Materials — (1) COVID-19 intubation checklist, (2) an intubation team handover document, and (3) Chelsea COVID-19 intubation experience. [file 8816729.f1.zip › 8816729.f1/Appendix 1 - COVID19 intubation checklist.pdf]

NON-THEATRE EMERGENCY INTUBATION CHECKLIST

COVID-19 intubation checklist

Chelsea and Westminster Hospital NHS
NHS Foundation Trust

PREPARATION – outside room

STEP 1: ALLOCATE ROLES

*****MAXIMUM 4 PEOPLE IN ROOM – all in full PPE*****

(enter name/ grade)

1. Airway (most senior anaesthetist) ____________ ☐
2. Airway assistant (ODP/ICU nurse) ____________ ☐
3. Drugs ____________ ☐
4. Team leader/2nd intubator *in room* ____________ ☐

plus 2 x Runners - *1 in antechamber* (in full PPE), *1 in corridor* ☐

ICU team aware of patient ☐
Transfer destination confirmed ☐

STEP 2: EQUIPMENT – leave COVID intubation trolley outside room

- Water's circuit with HME filter at mask ☐
- Airway adjuncts + suction + yankauer ☐
- i-gel (or alternative SAD, open & lubricate) ☐
- Video laryngoscope **1st line** with LoPro & Mac blades ☐
- Direct laryngoscope (if limited experience with VL) ☐
- Bougie + ETT x 2 (check cuff) with subglottic suction port ☐
- ETT ties + syringe + NG tube ☐
- Portable ventilator + tubing – checked + set to standby ☐
- FONA set (in COVID trolley) ☐
- Cannula x 2, dressing, chlorprep wipes + octopus connector ☐
- Scissors, pen (names on visors/gowns) + hand gel ☐

STEP 3: MONITORING

- ICU/theatre portable monitor (check battery) ☐
- SpO_2 (Set to audible) ☐
- ECG + BP set to cycle ☐
- Waveform Capnography ($ETCO_2$) ☐

STEP 4: DRUGS

Induction
Fentanyl/alfentanil/midazolam/ketamine

Paralysis
Rocuronium (**1st line**) /sugammadex *or* suxamethonium (if indicated)

Vasoactives + consider fluid pre-loading
Metaraminol/ephedrine/glyco/atropine/norad/adrenaline (10µg/ml)

Post intubation sedation *** obtain pumps + IV giving sets***
Propofol/fentanyl (**1st line**) *or* midazolam/morphine

RAPID SEQUENCE INTUBATION PLAN

Donn PPE – after all prep, with buddy, ideally in antechamber
FFP3 mask + visor + hat (ideally with neck cover) + long sleeve gown + fluid repellant gown + 2 x gloves

STEP 1: RISK ASSESSMENT - HAVE YOU CONSIDERED?

- Difficult laryngoscopy ☐
- Difficult cricothyroidotomy ☐
- Aspiration (stop NG feed/cricoid pressure required) ☐
- CVS instability (consider inotropes/fluid) ☐
- Obesity/Co-morbidities ☐
- Drug allergies ☐

STEP 2: AIRWAY MANAGEMENT - VERBALISE RSI PLAN (circle)

**Cricoid Pressure**	Locate cricothyroid membrane	
**Position**	Ramped	Head-up
**Other**	Aspirate NG, suction under pillow	

PREOXYGENATION *** no THRIVE/optiflow or NIV ***
Two-handed non-bagging technique with Water's circuit at minimum flow (e.g. ≤10L/min - lowest flow possible for adequate pre-ox)

PLAN A:
*** bagging/IPPV only after cuff inflation ***
1st line - videolaryngoscopy to limit contact with secretions
2nd line - direct laryngoscopy *if not trained in VL*

PLAN B/C:
i-gel/alternative SAD ± 2nd attempt at laryngoscopy
→ face-mask (two handed to maximise seal) with lowest possible flow

PLAN D:
Front of neck access (FONA)

*****Assign person to clean/restock trolley *****

Does anyone have any questions/concerns?

POST – INTUBATION

STEP 1: CONFIRM ETT POSITION

- ETT misting/$ETCO^2$ trace ☐
- Bilateral air entry – visual & manual confirmation (avoid stethoscope) ☐

STEP 2: CRITICAL CARE - VENTILATION

*****Avoid disconnections (clamp ETT if needed) *****

Optimise FiO_2, Tidal Volume + PEEP - liaise with ICU ☐

STEP 3: AIRWAY DOCUMENTATION

*** complete on Cerner Powerchart***

Intubation time & date

Indication for intubation & number of attempts

Location (circle) A&E AAU ICU Ward Theatres BICU

Intubation Grade

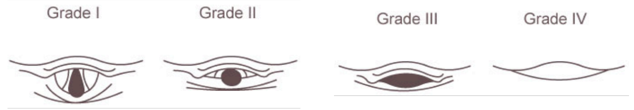

Final ETT size and length at teeth

**Video laryngoscopy**	Easy	Manageable	Difficult
**Direct laryngoscopy**	Easy	Manageable	Difficult
**Traumatic**	**Yes/No**		

Complications

Desat <88% 20% fall in BP SBP < 80mmHg or other

BEDSIDE DIFFICULT AIRWAY ALERT? YES/NO

CERNER DIFFICULT AIRWAY ALERT ? YES/NO

Version 1.2, March 2020
