# Supplementary material for: Emergency Tracheal Intubation in Patients with COVID-19: Experience from a UK Centre
Source: Anesthesiol Res Pract. 2020 Dec 10;2020:8816729. doi: 10.1155/2020/8816729 (PMC7729388; doi:10.1155/2020/8816729)
Supplement: Supplementary Materials — (1) COVID-19 intubation checklist, (2) an intubation team handover document, and (3) Chelsea COVID-19 intubation experience. [file 8816729.f1.zip › 8816729.f1/Appendix 2 - intubation team handover document.docx]

**Date:
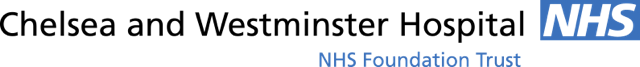
**

***COVID-19 AIRWAY TEAM 1***

**Roles (6-person team)**

1. Intubator (experienced anaesthetist):
2. Airway assistant (ODP/ICU nurse):
3. Drugs:
4. Team Leader (second airway doctor):
5. Runner 1:
6. Runner 2:

***Allocate roles & responsibilities at team huddle (7.45am & 7.45pm)***

***1) PPE - Ensure all PPE trained & mask fitted; make up PPE grab-bags***

***2) Airway Trolleys - Check intubation trolleys (and re-stock)***

***3) Transfer equipment - infusion pump, portable monitor & ventilator***

***4) Drugs - Check (and draw-up) intubation, emergency & sedation drugs***

**Date:
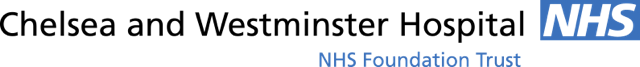
**

***COVID-19 AIRWAY TEAM 2***

**Roles (6-person team)**

1. Intubator (experienced anaesthetist):
2. Airway assistant (ODP/ICU nurse):
3. Drugs:
4. Team Leader (second airway doctor):
5. Runner 1:
6. Runner 2:

- ***Airway Team 2 also act as proning team and surgical trachy team (supported by Airway Team 1)***
- ***7^th^ person may be required for proning = ICU nurse looking after patient***
- ***Intubation/Airway trolley locations***
  - - ***3 x Main Theatres***
    - ***1 x Burns***
    - ***1 x Paeds***
    - ***1 x ICU***
    - ***1 x ED***
