# Supplementary material for: Emergency Tracheal Intubation in Patients with COVID-19: Experience from a UK Centre
Source: Anesthesiol Res Pract. 2020 Dec 10;2020:8816729. doi: 10.1155/2020/8816729 (PMC7729388; doi:10.1155/2020/8816729)
Supplement: Supplementary Materials — (1) COVID-19 intubation checklist, (2) an intubation team handover document, and (3) Chelsea COVID-19 intubation experience. [file 8816729.f1.zip › 8816729.f1/Appendix 3 - intubation experience survey.PDF]

Chelsea Covid-19 Intubation experience

We are gathering anonymous data surrounding human and procedural factors that may improve successful 1st pass intubation in the cohort of suspected/confirmed Covid-19 cases at Chelsea and Westminster Hospital. In particular we are looking for experiential data to supplement data gathered retrospectively from intubation records. We would be grateful if you could complete the below survey that should take no longer than 5 mins. Please complete this if you have been an intubator or made part of the intubation team. (Investigators: Patrick Ward, Chris Lockie, Jagdish Sokhi, Ajay Gandhi)

*Required

What is your current role? *

- ◯ ICU Consultant
- ◯ Anaesthetic Consultant
- ◯ Anaesthetic/ICU SpR
- ◯ Anaesthetic/ICU SHO
- ◯ Other:

What has been your role in Covid intubations? (please tick all that apply) *

- ☐ Primary intubator
- ☐ Team Leader
- ☐ Drugs
- ☐ Secondary Intubator
- ☐ Other

How would you rate your anxiety levels around intubation at the at the BEGINNING of the pandemic (Early March 2020)? *

	1	2	3	4	5	
no anxiety	○	○	○	○	○	high anxiety

How would you rate your anxiety levels around intubation currently (Early May 2020)? *

	1	2	3	4	5	
no anxiety	○	○	○	○	○	high anxiety

Which of the following environmental and procedural factors increased your levels of anxiety? (tick all that apply) *

- ☐ none
- ☐ PPE and personal safety
- ☐ Intubating in remote locations (i.e. out of theatre)
- ☐ Performance anxiety
- ☐ Use of a LocSSIP
- ☐ Using a prescribed "method"
- ☐ Using larger than normal diameter endotracheal tubes
- ☐ Different ETT with subglottic suction ports
- ☐ Using a video laryngoscope
- ☐ [illegible]

- [ ] unfamiliar setting
- [ ] unfamiliar team
- [ ] unfamiliar technique e.g. use of LMA to bag
- [ ] Other:

What patient factors made intubations most challenging?

- [ ] Hypoxia/desaturation
- [ ] CVS instability
- [ ] Increased airway oedema
- [ ] Difficulty in positioning,
- [ ] Prior use of CPAP,
- [ ] High BMI
- [ ] Time pressures

What elements do you find challenging about Enhanced PPE? (tick all that apply) *

- [ ] Physical restriction
- [ ] Reduced vision
- [ ] Inability to hear/communicate
- [ ] Temperature
- [ ] none

Did you deviate from your normal intubation practice? In what way? *

Your answer

In your usual ELECTIVE work, how often do you use a video laryngoscope? *

- ◯ <25% of intubations
- ◯ 25-50% of intubations
- ◯ 51-75% of intubations
- ◯ >75% of intubations

With respect to the Glidescope, which blades and adjuncts were you comfortable with using prior to Covid-19 pandemic? (tick all that apply) *

- ☐ VL- Mac
- ☐ VL- LoPro (hyperangulated)
- ☐ Stylet
- ☐ Bougie

In how many intubations did you take the role of TEAM LEADER? *

- ◯ 0
- ◯ 1-2
- ◯ 3-4
- ◯ 5-6
- ◯ 7-8
- ◯ 9-10
- ◯ >10

In how many intubations did you take the role of PRIMARY INTUBATOR? *

- 0
- 1-2
- 3-4
- 5-6
- 7-8
- 9-10
- >10

How many intubations were you directly involved in? *

- 0
- 1-2
- 3-4
- 5-6
- 7-8
- 9-10
- >10

Submit

Never submit passwords through Google Forms.

This content is neither created nor endorsed by Google. Report Abuse - Terms of Service - Privacy Policy

Google Forms
